# Supplementary material for: Long-Term SARS-CoV-2-Specific Humoral and T Cell Responses after the BNT162b2 or BBIBP-CorV Booster and the Incidence of Breakthrough Infections among Healthcare Workers
Source: Vaccines (Basel). 2023 Dec 19;12(1):3. doi: 10.3390/vaccines12010003 (PMC10819931; doi:10.3390/vaccines12010003)
Supplement: Supplementary file 1 [file vaccines-12-00003-s001.zip › vaccines-2733474-supplementary.pdf]

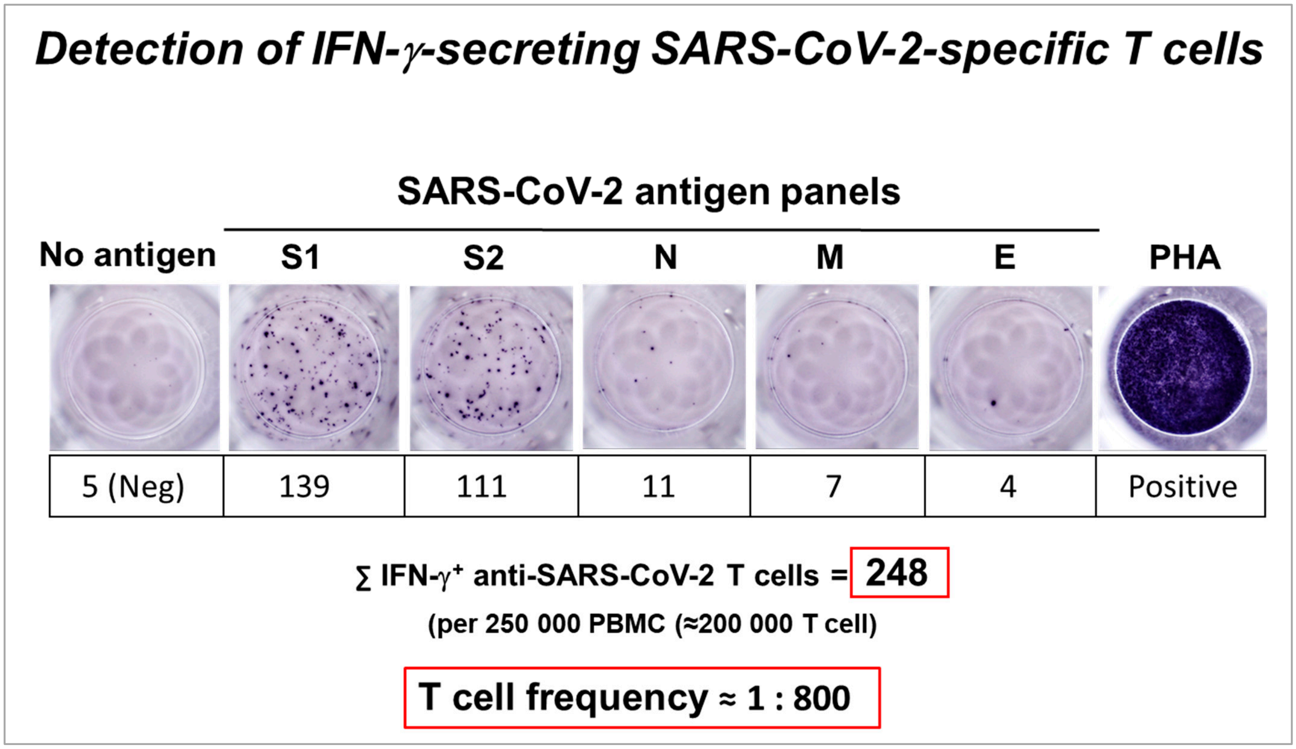

Figure S1. Representative ELISpot assay result of one study participant. The microtiter wells were photographed using the AID vSPOT ELISPOT reader. The IFNγ-producing effector T cells specific to different antigens were quantified by counting the spots in each well. The cumulative spot-forming units (SFUs) per 250,000 PBMCs were calculated as the total number of T-spots for Spike (S1 and S2), Nucleocapsid (N), Membrane (M), and Envelope (E) antigens minus the background for each antigen.
